# Metastatic HPV-Related Multiphenotypic Sinonasal Carcinoma: A Case Report and Review of the Literature

**DOI:** 10.1055/a-2773-6796

**Published:** 2026-03-27

**Authors:** Rachel Stemme, Wassim Najjar, Tanguy Y. Seiwert, Harry Quon, Lisa M. Rooper, Debraj Mukherjee, Nicholas R. Rowan

**Affiliations:** 1 Department of Otolaryngology-Head and Neck Surgery, Johns Hopkins University School of Medicine, Baltimore, Maryland, United States; 2 Department of Oncology, Sidney Kimmel Comprehensive Cancer Center, Johns Hopkins University School of Medicine, Baltimore, Maryland, United States; 3 Department of Radiation Oncology and Molecular Radiation Sciences, Johns Hopkins University School of Medicine, Baltimore, Maryland, United States; 4 Department of Pathology, Johns Hopkins University School of Medicine, Baltimore, Maryland, United States; 5 Department of Neurosurgery, Johns Hopkins University School of Medicine, Baltimore, Maryland, United States

**Keywords:** HPV, multiphenotypic sinonasal carcinoma, human papillomavirus, sinonasal cancer

## Abstract

**Background:**

Human papillomavirus (HPV)–related multiphenotypic sinonasal carcinoma (HMSC) is a rare neoplasm characterized by a paradoxical clinical course. Despite its high-grade histologic features, HMSC exhibits an unexpectedly indolent clinical behavior, marked by frequent local recurrences but limited metastatic potential. We report the first case of HMSC metastasis to the liver with the earliest known distant progression.

**Case Report:**

A 53-year-old male who presented with unilateral epistaxis was found to have T4aN0M0 HPV35-positive HMSC of the left maxillary sinus, with erosion into the orbital floor. He underwent induction chemotherapy, endoscopic resection with negative margins, and adjuvant chemoradiation. He did well until surveillance PET-CT at 10 months revealed asymptomatic hepatic metastases confirmed by biopsy. He was treated with transarterial chemoembolization and pembrolizumab before developing locoregional recurrence involving the orbital apex and cavernous sinus 5 months later.

**Conclusion:**

Several case reports demonstrate HMSC's potential for aggressive behavior, including intracranial extension and rapid recurrence. However, distant metastases (DM) are rare, with only three documented cases of pulmonary metastases and one digital metastasis, occurring years after initial treatment. We present the first reported case of HMSC with metastasis to the liver, marking the earliest known distant recurrence. This case challenges the current paradigm by highlighting HMSC's potential for aggressive systemic progression. Molecularly, this tumor harbored high-risk HPV35, a rarely reported subtype. Despite HMSC's typically indolent course, this case underscores the importance of vigilant surveillance to detect early DM and guide therapeutic management.

## Introduction

Human papillomavirus (HPV)-related multiphenotypic sinonasal carcinoma (HMSC), newly described in 2013, is characterized by high-grade histopathological features. 1 HMSC typically exhibits invasive local growth with a tendency for local recurrence. 2 Despite its locally aggressive behavior, HMSC is considered to have a relatively indolent clinical course with limited metastatic potential and a more favorable prognosis compared with other aggressive sinonasal malignancies. 3

## Case Report

**Fig. 1:**
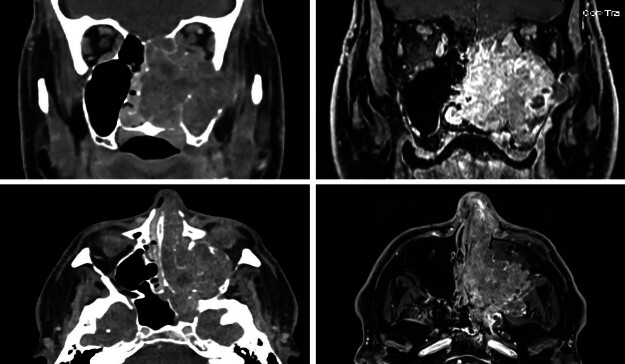
Preoperative computed tomography (CT) and magnetic resonance imaging (MRI) of 8.1 cm × 4.8 cm × 6.5 cm (AP × TV × CC) left HPV-related multhiphenotypic sinonasal carcinoma (HMSC) with extensive bony destruction.

**Fig. 2:**
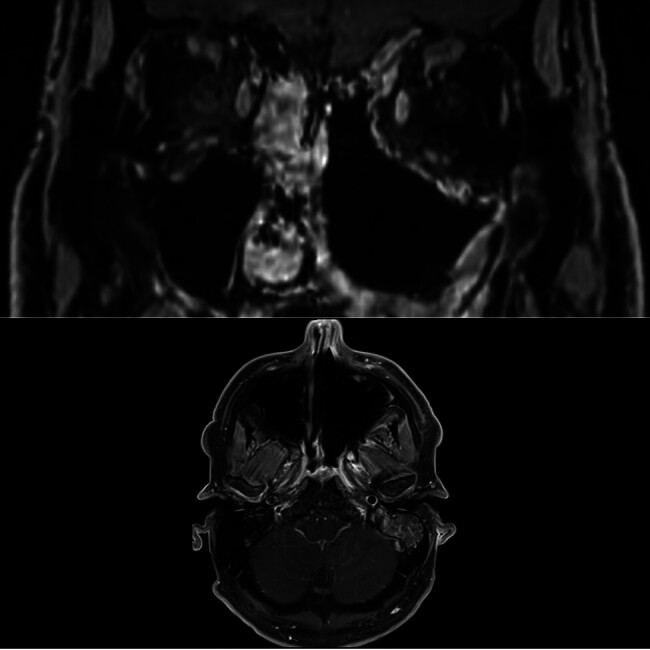
Postsurgical resection and adjuvant chemoradiotherapy, magnetic resonance imaging (MRI) of left HPV-related multiphenotypic carcinoma (HMSC) without evidence of residual or recurrent tumor.

**Fig. 3:**
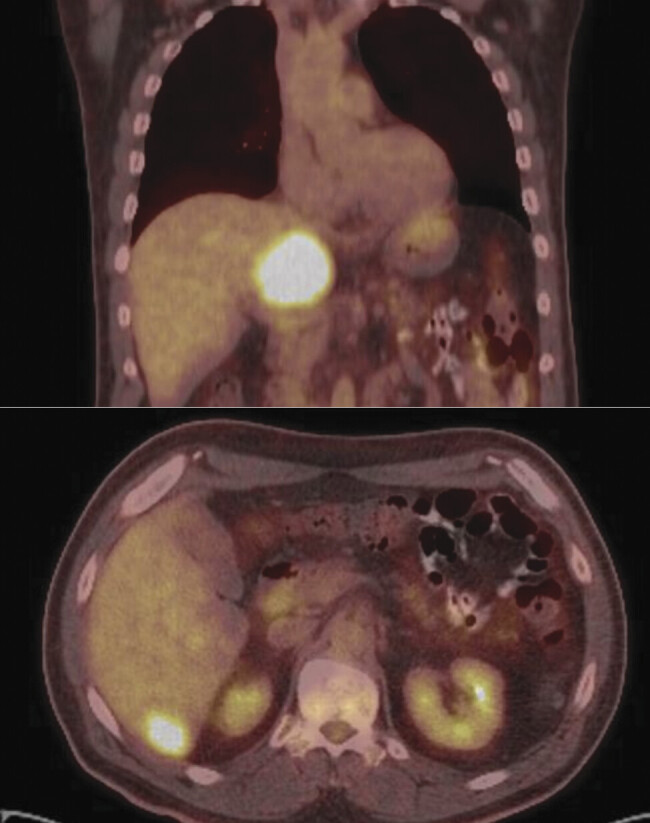
Positron emission tomography/computed tomography (PET/CT) obtained 10 months posttreatment showing FDG-avid 7.7 cm × 5.7 cm caudate lobe mass and scattered FDG-avid liver lesions.

A 53-year-old male with a history of daily cigar-smoking presented with 6-months of progressive unilateral epistaxis, nasal congestion, and facial pressure. Anterior rhinoscopy revealed a mass obstructing the left nasal airway. CT imaging revealed a 5 cm × 8 cm expansile lesion occupying the left maxillary sinus with erosion of the left orbital floor ( Fig. 1 ). A biopsy and imaging were diagnostic of T4aN0M0 HMSC. Immunohistochemistry demonstrated diffuse positivity for p16, and in situ hybridization confirmed the presence of high-risk HPV RNA. Treatment included induction chemotherapy (carboplatin/paclitaxel), with moderate treatment response, followed by orbital-sparing endoscopic resection with negative margins, and adjuvant chemoradiotherapy (cisplatin) to a cumulative dose of 7,500 cGy. Initial surveillance found no evidence of recurrent or locoregional disease ( Fig. 2 ). Due to insurance difficulties, a PET-CT was delayed until 10 months post-treatment, which revealed an asymptomatic liver mass and multiple liver lesions ( Fig. 3 ). A biopsy confirmed metastatic HMSC. Subsequently, the patient underwent three transarterial chemoembolization procedures with doxorubicin and immunotherapy with pembrolizumab. Despite 5 months of continuous treatment, he has experienced progressive liver disease and locoregional recurrence in the cavernous sinus.

## Literature Review

The “paradoxical” nature of HMSC was first highlighted in a 49-patient case series, where one-third had local recurrence, while distant metastases (DM) occurred in two cases. 2 One patient had DM in the lung and finger 144 months after surgery and radiotherapy, and another had DM in the lung 96 months after surgery. A review of 79 HMSC cases reinforced its typically indolent clinical course, identifying no regional metastases and only one tumor-related death. 3 However, in 2019, early distant metastasis to the lung was reported, occurring 23 months after treatment. 4

In the United States, HPV-related sinonasal neoplasms are increasingly prevalent with significant prognostic implications. 5 6 7 HMSC is most often associated with HPV type 33, but HPV types 35, 56, and 16 have also been reported. 3 This is notably different from HPV-related oropharyngeal SCC, where HPV type 16 predominates.

More aggressive presentations of HMSC with intracranial extension and rapid local recurrence complicate the establishment of a definitive treatment strategy. 8 9 10 Although typical treatment of HMSC has consisted of surgery and radiotherapy, the role of induction chemotherapy may also be considered and is an area of interest in the management of sinonasal SCC more broadly. 11 12 13

We report the first case of hepatic metastasis from HMSC and the earliest known instance of distant disease progression. This case demonstrates that HMSC can exhibit aggressive systemic behavior, contradicting current understanding of its typically indolent course. These findings underscore the importance of continued monitoring in HPV-related disease and support investigating induction chemotherapy or ctDNA surveillance for patients with HMSC.
